# Work-related asthma in adults with severe asthma from the Korean Severe Asthma Registry (KoSAR)^☆^

**DOI:** 10.1016/j.waojou.2024.100903

**Published:** 2024-05-22

**Authors:** Youngsoo Lee, Sun-Kyung Lee, So-Young Park, Min-Hye Kim, Sung-Yoon Kang, Ga-Young Ban, Young-Hee Nam, Joo-Hee Kim, Chin Kook Rhee, Woo-Jung Song, Jae-Woo Kwon, Taehoon Lee, So Ri Kim, Heung-Woo Park, You Sook Cho, Young-Il Koh, Kwang-Ha Yoo, Byung-Jae Lee, Ho Joo Yoon, Hae-Sim Park, Sang-Heon Kim

**Affiliations:** a Department of Allergy and Clinical Immunology, Ajou University School of Medicine, Suwon, South Korea; b Department of Mathematics, College of Natural Sciences, Hanyang University, Seoul, South Korea; c Division of Pulmonary Medicine and Allergy, Department of Internal Medicine, Hanyang University College of Medicine, Seoul, South Korea; d Department of Internal Medicine, Chung-Ang University College of Medicine, Gwangmyeong, South Korea; e Department of Internal Medicine, Ewha Woman's University College of Medicine, Seoul, South Korea; f Department of Internal Medicine, Gachon University Gil Medical Center, Incheon, South Korea; g Department of Pulmonary, Allergy and Critical Care Medicine, Kangdong Sacred Heart Hospital, Hallym University College of Medicine, Seoul, South Korea; h Department of Internal Medicine, Dong-A University College of Medicine, Busan, South Korea; i Department of Internal Medicine, Hallym University College of Medicine, Anyang, South Korea; j Division of Pulmonary and Critical Care Medicine, Department of Internal Medicine, Seoul St Mary's Hospital, College of Medicine, The Catholic University of Korea, Seoul, South Korea; k Department of Allergy and Clinical Immunology, Asan Medical Center, University of Ulsan College of Medicine, Seoul, South Korea; l Department of Internal Medicine, Kangwon National University School of Medicine, Chuncheon, South Korea; m Division of Pulmonary and Critical Care Medicine, Department of Internal Medicine, Ulsan University Hospital, University of Ulsan College of Medicine, Ulsan, South Korea; n Division of Respiratory Medicine and Allergy, Department of Internal Medicine, Chonbuk National University Medical School, Jeonju, South Korea; o Department of Internal Medicine, Seoul National University College of Medicine, Seoul, South Korea; p Division of Allergy, Asthma and Clinical Immunology, Department of Internal Medicine, Chonnam National University Medical School, Chonnam National University Hospital, Gwangju, South Korea; q Division of Pulmonary, Allergy and Critical Care Medicine, Department of Internal Medicine, Konkuk University School of Medicine, Seoul, South Korea; r Department of Medicine, Sungkyunkwan University School of Medicine, Seoul, South Korea

**Keywords:** Work-related asthma, Severe asthma, Quality of life, Depression, Anxiety

## Abstract

**Background:**

Exposure to allergens or irritants in the workplace may affect asthma control and the quality of life (QoL) of patients with asthma.

**Objective:**

To examine the prevalence and characteristics of work-related asthma (WRA) in adult patients with severe asthma.

**Methods:**

We analyzed data from the Korean Severe Asthma Registry (KoSAR), which is a nationwide multicenter observational study on severe asthma in Korea. Severe asthma was defined according to the American Thoracic Society (ATS) and the European Respiratory Society (ERS) guidelines. WRA was identified on the basis of asthma symptom aggravation at the workplace, as indicated by responses to a structured questionnaire. We compared the demographic and clinical characteristics and QoL between adult patients with severe asthma and WRA and those without WRA.

**Results:**

Among 364 patients with severe asthma who were employed at the time of enrollment, 65 (17.9%) had WRA. There were no significant differences in age, sex, obesity, or smoking history between the WRA and non-WRA groups. However, individuals with WRA exhibited a higher prevalence of anxiety (7.7% vs 2.4%, P = 0.046) and depression (12.3% vs 3.7%, P = 0.010) than those without. The levels of asthma control, lung function, and frequency of asthma exacerbations were similar between the two groups, but patients with WRA reported lower QoL, as determined by the Quality of Life Questionnaire for Adult Korean Asthmatics (56.6 ± 14.6 vs. 63.5 ± 13.9, P < 0.001).

**Conclusion:**

Patients with severe asthma and WRA are more likely to experience anxiety and depression and have lower QoL than those without WRA.

## Introduction

Asthma, a chronic inflammatory disease affecting the lower airways, is characterized by airway reversibility and hyperresponsiveness. Although patients with severe asthma account for only 3%–5% of all asthmatics, more than half of the total medical expenses of asthmatics are attributed to severe asthma given its poor symptom control, hospitalization due to asthma exacerbation (AE), and high morbidity and mortality.^1^, ^2^, ^3^ In patients with severe asthma, asthmatic symptoms are more likely to be aggravated by multiple factors such as upper respiratory infection, smoking, air pollution, and environmental and occupational exposure.^4^

Work-related asthma (WRA) is a phenotype of asthma that is worsened due to the environment of the workplace.^5^ WRA is a broad term encompassing 2 types, namely occupational asthma (OA) and work-exacerbated asthma (WEA). Although patients with WRA comprise 10%–30% of all asthmatics, they are more likely to have a higher probability of uncontrolled symptoms and frequent AEs than those without WRA (non-WRA).^6^ Moreover, compared to patients without WRA, those with WRA require significantly more healthcare resources and higher medical costs.^6^, ^7^, ^8^ Despite the clinical and economic impact of WRA, only a few studies have characterized patients with WRA, with most focusing on OA.^9^, ^10^, ^11^ Furthermore, no previous study has reported how many patients with severe asthma accompany WRA and whether the presence of WRA affects asthma control, AEs, and quality of life (QoL) in patients with severe asthma, although WRA has been proposed as one of the aggravating or causative factors of severe asthma.^12^, ^13^, ^14^ Therefore, the aim of this study was to identify the prevalence and clinical characteristics of patients with severe asthma with WRA compared to those without WRA in the Korean Severe Asthma Registry (KoSAR), a national registry of patients with severe asthma.

## Methods

### Study subjects and definitions

The KoSAR was established in 2009 by the Working Group on Severe Asthma of the Korean Academy of Asthma, Allergy, and Clinical Immunology (KAAACI). The study network currently comprises 39 university hospitals in Korea.^15^, ^16^, ^17^

Adult patients diagnosed with severe asthma according to the European Respiratory Society (ERS)/American Thoracic Society (ATS) severe asthma definition were consecutively recruited in the KoSAR. Severe asthma was defined as “asthma that requires treatment with high-dose inhaled corticosteroids (ICS) plus a second controller (and/or systemic corticosteroids) to prevent it from becoming “uncontrolled” or that remains “uncontrolled” despite this therapy”.^18^ Patients with severe bronchiectasis, tuberculosis-destroyed lungs, interstitial lung disease, severe congestive heart failure, or any diseases other than asthma that were treated with oral corticosteroid (OCS) maintenance were excluded. We analyzed data from patients who participated in the KoSAR from January 2010 to April 2022.

The patients’ demographics (age, sex, body mass index [BMI], smoking history), asthma history (age at symptom onset, age at diagnosis, age at treatment initiation, duration of treatment, asthma medications), and comorbidities (allergic rhinitis, allergic conjunctivitis, atopic dermatitis, chronic sinusitis, hypertension, osteoporosis, anxiety disorder, depression) were collected using the KoSAR questionnaires. Laboratory parameters (complete blood cell counts, total IgE levels, and sputum cell counts), skin prick test results for common inhalant allergens, lung function (forced expiratory volume in 1 s [FEV1], forced vital capacity [FVC], and FEV1/FVC), and fraction of exhaled nitric oxide (FeNO) were gathered. Atopy was defined as a positive skin prick test or a positive serum specific IgE result for at least one common inhalant allergen.

Occupational information was collected for the participants’ current job, job duration, aggravation of asthmatic symptoms in the workplace, jobs before being diagnosed with asthma, and exposure to specific substances. WRA was defined if a patient responded to the questionnaire that asthmatic symptoms were provoked or aggravated in their workplace; if not, the patient was defined as not having WRA.

The asthma control status was evaluated according to the GINA criteria (controlled, partly controlled, and uncontrolled) and the Asthma Control Test (ACT). The participants’ QoL was evaluated using the Quality of Life Questionnaire in Adult Korean Asthmatics (QLQAKA).^19^ AE was classified into 2 categories: OCS burst for ≥3 consecutive days or unscheduled visits within a year before baseline. In addition, information on unscheduled healthcare use due to AE was collected and classified into four categories: outpatient department visit, emergency department visit, hospitalization, and intensive care unit admission.

This study was approved by the Institutional Review Board of each participating hospital, and informed consent was obtained from the study participants (IRB number: AJOUIRB-OBS-2022-171).

### Statistical analysis

Data are presented as counted numbers and their corresponding percentages for categorical variables and the mean ± standard deviation for continuous variables. The categorical variables were compared between the groups by χ^2^ test or Fisher's exact test, and continuous variables were compared using the independent *t*-test or Mann–Whitney U test. All statistical analyses were performed using SAS 9.4 (SAS Institute, Cary, NC, USA). A two-sided *P*-value <0.05 was considered statistically significant.

## Results

**Fig. 1:**
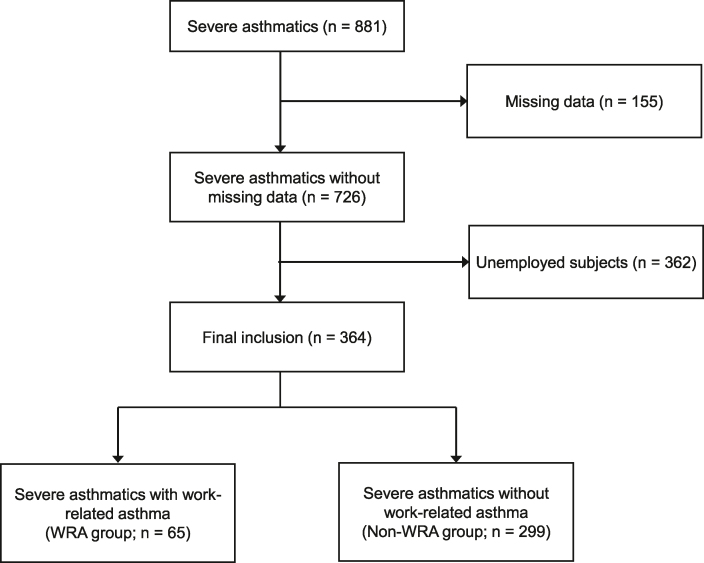
Flowchart of patient inclusion.

**Fig. 2:**
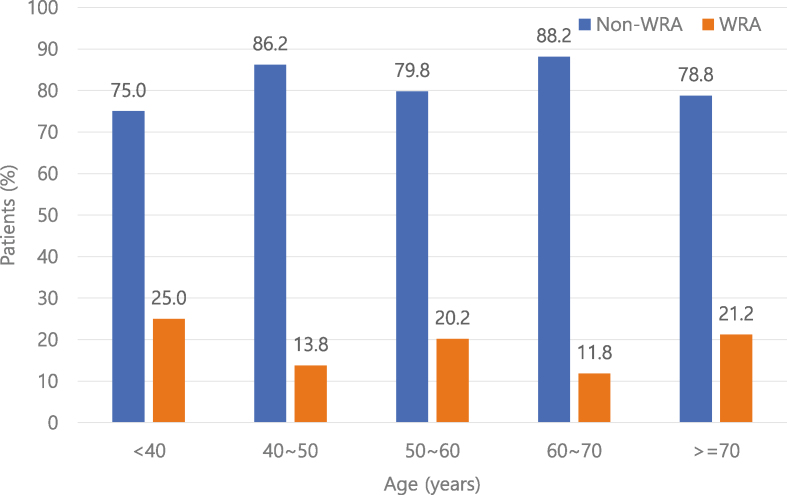
Proportions of patients in the WRA and non-WRA groups by age.

A total of 881 patients with severe asthma were recruited in the KoSAR (Fig. 1). After excluding 155 patients with missing data and 362 unemployed patients at baseline, 364 patients with severe asthma were included in the analysis. A considerable proportion of patients (65, 17.9%) were identified as having WRA and were thus categorized into the WRA group. In contrast, 299 (82.1%) patients were determined not to have WRA and were thus placed in the non-WRA group. The proportion of patients with WRA by age was as follows: 25%, <40 years old; 13.8%, ≥40 and <50 years old; 20.2%, ≥50 and <60 years old; 11.8%, ≥60 and <70 years old; and 21.2%, ≥70 years old (Fig. 2). The workplace-exposed materials that caused AE were isocyanate (12.3%), welding flux (7.7%), reactive dye (1.5%), epoxy (7.7%), nickel compounds (1.5%), drug aerosol (4.6%), flour (7.7%), grain powder (9.2%), and other chemicals (30.8%).

The baseline demographic and clinical characteristics of the WRA and non-WRA groups are summarized in Table 1. The age at recruitment, sex, BMI, and smoking history were comparable between the groups. The age at asthma symptom onset, age at asthma diagnosis, age at asthma treatment initiation, and duration of asthma treatment were also similar. The patients in the WRA group demonstrated a substantially higher prevalence of anxiety, with 7.7% reporting symptoms compared to only 2.4% in the non-WRA group (*P* = 0.046). Furthermore, an even more pronounced difference was observed in the prevalence of depression, with 12.3% of the WRA group reporting depression compared to only 3.7% of the non-WRA group (*P* = 0.010). The proportions of the other comorbidities, including allergic rhinitis, allergic conjunctivitis, atopic dermatitis, chronic sinusitis, hypertension, and osteoporosis, did not exhibit significant differences between the two groups.

**Table 1:** Baseline demographic characteristics, asthma history, and comorbidities of the WRA and non-WRA groups.

	WRA group (n = 65)	Non-WRA group (n = 299)	*P-value*
Age, years	50.1 ± 15.2	52.0 ± 13.7	0.306
Male	38 (58.5)	191 (63.9)	0.412
Body mass index^a^	24.4 ± 3.5	24.4 ± 4.1	0.975
Underweight	2 (3.1)	18 (6.0)	0.536
Normal	22 (33.9)	89 (29.8)	
Overweight	20 (30.8)	77 (25.8)	
Obesity	21 (32.3)	115 (38.5)	
Smoking history			
Never smoker	35 (53.9)	142 (47.7)	0.591
Ex-smoker	22 (33.9)	121 (40.6)	
Current smoker	8 (12.3)	35 (11.7)	
Asthma history, years			
Age at asthma symptom onset (n = 341)	37.7 ± 16.1	38.1 ± 15.2	0.827
Age at asthma diagnosis (n = 339)	40.0 ± 16.0	39.7 ± 14.7	0.872
Age at asthma treatment initiation (n = 357)	39.5 ± 15.8	41.5 ± 16.4	0.374
Duration of asthma treatment (n = 357)	10.1 ± 9.7	10.8 ± 11.5	0.667
Comorbidities			
Atopy (n = 183)	17 (48.6)	72 (48.7)	0.993
Allergic rhinitis (n = 358)	48 (75.0)	212 (72.1)	0.638
Allergic conjunctivitis (n = 360)	8 (12.3)	33 (11.2)	0.797
Atopic dermatitis (n = 362)	10 (15.4)	23 (7.7)	0.053
Chronic sinusitis (n = 357)	26 (40.6)	97 (33.1)	0.252
Hypertension (n = 363)	15 (23.1)	61 (20.5)	0.640
Osteoporosis (n = 362)	1 (1.5)	16 (5.4)	0.328
Anxiety disorder (n = 362)	5 (7.7)	7 (2.4)	0.046
Depression (n = 362)	8 (12.3)	11 (3.7)	0.010

Values are presented as the mean ± standard deviation or number (%).

WRA, work-related asthma.

a Obesity ≥25; 23 ≤ overweight <25; 18.5 ≤ normal <23); underweight <18.5

The baseline laboratory findings and lung function were compared between the 2 groups (Table 2). Blood leukocytes (*P* = 0.168), the percentages of blood neutrophils (*P* = 0.759) and eosinophils (*P* = 0.456), total IgE levels (*P* = 0.265), the proportion of atopic patients (*P* = 0.993), and FeNO levels (*P* = 0.396) were comparable between the 2 groups. The sputum cell counts (%), including eosinophils (*P* = 0.785) and neutrophils (*P* = 0.939), were also comparable between the groups, as were the lung function variables, including FEV1 (*P* = 0.495), FEV1% (*P* = 0.700), FVC (*P* = 0.642), FVC% (*P* = 0.234), and FEV1/FVC (*P* = 0.653).

**Table 2:** Baseline laboratory findings and lung function of the WRA and non-WRA groups.

	WRA group (n = 65)	Non-WRA group (n = 299)	*P-value*
WBC, × 10^3^/μL (n = 325)	8.7 ± 3.0	8.1 ± 2.7	0.168
Blood eosinophils, % (n = 320)^a^	3.4 (2.0–7.7)	3.7 (1.2–7.3)	0.456
Blood eosinophils, /μL (n = 320)^a^	299.2 (141.1–660.0)	261.1 (99.5–531.4)	0.238
Blood neutrophils, % (n = 255)	56.6 ± 11.8	57.2 ± 12.9	0.759
Total IgE, IU/mL (n = 109)^a^	147.6 (81.3–777.0)	366.8 (138.2–790.9)	0.265
FENO, ppb (n = 141)^a^	33.0 (17.0–114.0)	36.5 (21.0–60.0)	0.396
Sputum eosinophil, % (n = 97)^a^	5.0 (1.0–24.0)	5.0 (1.0–50.7)	0.785
Sputum neutrophil, % (n = 98)^a^	56.5 (15.0–85.5)	38 (15.3–85.0)	0.939
FEV1, L	2.2 ± 0.8	2.1 ± 0.81	0.495
FEV1, %	68.5 ± 20.1	67.2 ± 25.4	0.700
FVC, L	3.2 ± 0.9	3.1 ± 1.0	0.642
FVC, %	81.7 ± 12.9	79.3 ± 14.7	0.234
FEV1/FVC	67.6 ± 16.6	66.7 ± 14.7	0.653

Values are presented as the mean ± standard deviation, number (%), or median with interquartile range.

FENO, fraction of exhaled nitric oxide; FEV1, forced expiratory volume in 1 s; FVC, forced vital capacity; WRA, work-related asthma.

a Mann–Whitney U test

The proportions of patients with controlled (20.7% vs. 23.6%), partly controlled (50.0% vs. 45.6%), and uncontrolled (29.3% vs. 30.8%) status were comparable between the WRA and non-WRA groups, respectively (*P* = 0.819) (Table 3), as were the ACT scores (17.6 ± 4.8 vs. 18.2 ± 5.1, *P* = 0.373). However, the WRA group showed a significantly lower QLQAKA score than the non-WRA group (56.6 ± 14.6 vs. 63.5 ± 13.9, *P* < 0.001). Each QLQAKA item and its corresponding score in the WRA and non-WRA groups are described in Table 4. The patients in the WRA group reported a significantly increased frequency of emotional stress due to asthma (*P* < 0.001); aggravated asthma symptoms due to weather, temperature changes, or air pollution (*P* < 0.001); aggravated asthma symptoms due to indoor dust or hazy air (*P* < 0.001); sleep disturbance due to cough or dyspnea (*P* = 0.003); limitations in occupational activities due to asthma (*P* = 0.004); and cough (*P* = 0.009).

**Table 3:** Asthma control status, ACT score, and quality of life (measured by Quality of Life Questionnaire for Adult Korean Asthmatics) of the WRA and non-WRA groups.

	WRA group (n = 65)	Non-WRA group (n = 299)	*P-value*
Asthma control status, n (%)			0.819
Controlled	12 (20.7)	65 (23.6)	
Partly controlled	29 (50.0)	126 (45.6)	
Uncontrolled	17 (29.3)	85 (30.8)	
ACT score	17.6 ± 4.8	18.2 ± 5.1	0.373
QLQAKA score	56.6 ± 14.6	63.5 ± 13.9	<0.001

Values are presented as the mean ± standard deviation or number (%).

ACT, asthma control test; QLQAKA, Quality of Life Questionnaire for Adult Korean Asthmatics; WRA, work-related asthma

**Table 4:** Scores of the questions in the Quality of Life Questionnaire for Adult Korean Asthmatics (QLQAKA) of the WRA and non-WRA groups.

Question	WRA group (n = 65)	Non-WRA group (n = 299)	*P-value*
1. Chest discomfort	3.4 ± 1.1	3.7 ± 1.0	0.046
2. Concern about suffering from asthma exacerbation	3.2 ± 1.4	3.7 ± 1.4	0.019
3. Dyspnea due to asthma	3.3 ± 1.0	3.7 ± 1.2	0.017
4. Asthma symptom provoked by smoke or a pungent smell	3.0 ± 1.6	3.3 ± 1.6	0.094
5. Wheezing	3.4 ± 1.4	3.8 ± 1.2	0.022
6. Cough	3.1 ± 1.1	3.5 ± 1.0	0.009
7. Emotional stress due to asthma	3.0 ± 1.4	3.7 ± 1.3	<0.001
8. Sleep disturbance due to cough or dyspnea	3.6 ± 1.3	4.1 ± 1.1	0.003
9. Aggravated asthma symptoms by weather, temperature changes or air pollution	3.5 ± 1.4	4.1 ± 1.2	<0.001
10. Concern about asthma treatment	3.3 ± 1.3	3.6 ± 1.3	0.129
11. Sputum	2.6 ± 1.3	3.0 ± 1.3	0.036
12. Asthma symptoms aggravated by indoor dust or hazy air	3.2 ± 1.1	3.8 ± 1.2	<0.001
13. Limitations in strenuous physical activities due to asthma	3.1 ± 1.1	3.5 ± 1.2	0.020
14. Limitations in light daily activities due to asthma	3.7 ± 1.1	4.0 ± 1.0	0.038
15. Limitations in social activities due to asthma	3.8 ± 1.3	4.1 ± 1.0	0.118
16. Limitations in occupational activities due to asthma	3.8 ± 1.1	4.2 ± 0.9	0.004
17. Limitations in all daily activities due to asthma	3.5 ± 1.0	3.9 ± 0.9	0.013

Values are presented as the mean ± standard deviation.

Scores for each question range from 1 (almost all the time) to 5 (not at all).

WRA, work-related asthma

The frequency of AEs during the previous year was compared between the WRA and non-WRA groups (Table 5). The proportions of patients who used OCS bursts at least once (50.0% vs. 37.4%, *P* = 0.074) and who used ≥3 OCS bursts (22.4% vs. 18.1%, *P* = 0.452) were comparable between the WRA and non-WRA groups, respectively. Other outcomes were also similar between the WRA and non-WRA groups, including unscheduled outpatient visits (23.1% vs. 21.1%, *P* = 0.721), emergency department visits (18.5% vs. 15.7%, *P* = 0.587), hospitalization (21.5% vs. 15.1%, *P* = 0.198), and intensive care unit admissions due to AE (0% vs. 1.0%, *P* = 1.000).

**Table 5:** Asthma exacerbations (steroid burst, unscheduled visit, admission) of the WRA and non-WRA groups within a year before baseline.

	WRA group (n = 65)	Non-WRA group (n = 299)	*P-value*
Steroid burst			
Steroid burst treatment ≥3 days (n = 306)	30/60 (50.0)	92/246 (37.4)	0.074
Number of steroid bursts ≥3 days (n = 301)			
< 3	45/58 (77.6)	199/243 (81.9)	0.452
≥ 3	13/58 (22.4)	44/243 (18.1)	
Unscheduled healthcare use			
Outpatient department visit (n = 364)	15 (23.1)	63 (21.1)	0.721
Emergency department visit (n = 364)	12 (18.5)	47 (15.7)	0.587
Hospitalization (n = 364)	14 (21.5)	45 (15.1)	0.198
Intensive care unit admission (n = 363)	0 (0.0)	3 (1.01)	1.000

Values are presented as number (%).

WRA, work-related asthma

## Discussion

To the best of our knowledge, this is the first study on the prevalence and clinical characteristics of WRA in patients with severe asthma. The patients in the WRA group demonstrated a higher prevalence of anxiety disorder and depression, with a poorer QoL than those in the non-WRA group. However, the inflammatory profiles (blood/sputum eosinophils, total IgE level, and FeNO), lung function, and asthma control status were not significantly different between the 2 groups. The frequency and number of OCS bursts in the previous year of baseline were also comparable. These findings suggest that severe asthmatics with WRA may suffer from anxiety disorder, depression, and poor QoL.

Despite the relative wealth of data on general asthma, research into the prevalence of WRA specifically within severe asthmatics has been limited. Our findings revealed that 17.9% of patients with severe asthma were affected by WRA. Previous studies have shown inconsistent results regarding the prevalence of WRA in patients with asthma. For instance, in Ulsan, a highly industrialized city in Korea, the prevalence stood at 17%, which aligned with the results from other nations.^20^, ^21^, ^22^ Moreover, an investigation in Spain found that 32.9% of adult asthmatics had WRA, which surpassed the percentage reported in the current study.^23^ Various factors, including study design, regional differences, and participant demographics, may have contributed to these discrepancies. Crucially, while the aforementioned studies assessed the prevalence of WRA within general asthma populations, our study suggests that the impact of WRA on patients with severe asthma mirrors the impact on general asthmatic population. Given the potential pathophysiological implications of work-related exposures, further evaluation on the prevalence of WRA among patients with severe asthma is imperative.^14^

Interestingly, among patients older than 70 years, the proportion of patients with WRA was the second highest and as high as that in patients younger than 40 years. The Organization for Economic Cooperation and Development (OECD) defines working age as 15–64 years and the elderly population as people aged ≥65 years.^24^ According to the European Union, 4.9 million people older than 65 years were reported to be in employment in 2014, which was a marked increase of 48% from 3.3 million in 2004. The increase in the elderly population is a common phenomenon worldwide, and the number of elderly people is estimated to nearly triple by 2050, which is 16% of the global population.^25^ As a result, in Korea, 2 out of 5 people are estimated to be aged ≥65 years in 2050. The prevalence of elderly asthma is also expected to increase, which is currently reported to be between 4% and 13%.^26^, ^27^, ^28^ Besides, WRA is more significant in the elderly population than that in younger populations in terms of morbidities. In a cross-sectional study encompassing 14.8 million ever-employed adults with current asthma, those with WRA showed a higher probability of having cardiovascular diseases than those without.^29^ Nevertheless, the prevalence and disease burden of WRA in elderly asthmatics remain largely uncertain. Further studies are required to establish the prevalence of WRA in elderly asthmatics because the number of elderly asthmatics in employment is likely to increase continuously. Another notable demographic finding is that there was no sex difference between the WRA and non-WRA groups, although the prevalence of WRA and OA has been reported to be higher in males than in females in previous studies. The jobs and work may be different between male and female patients with severe asthma, but the impact of WRA and OA seems substantial in both sex groups.^30^

Although objective parameters such as lung function provide valuable insights into the physiological aspects of asthma, patient-reported outcomes, such as QoL and psychological distress, are equally crucial for a comprehensive understanding of the impact of asthma. In our study, patients in the WRA group had a lower QoL, as measured by the questionnaire, and exhibited a higher degree of anxiety and depression than those in the non-WRA group. Asthmatic symptoms are known to impose significant psychological burdens on patients, with several studies reporting a higher incidence of both anxiety and depression in asthmatics than in non-asthmatics.^31^, ^32^, ^33^, ^34^, ^35^, ^36^, ^37^ Although variations in the prevalence of psychological disorders among asthmatics have been observed, studies have shown significant percentages, with one study reporting that 36.9% and 11% of outpatients with asthma had anxiety and depression, respectively. Cases of severe asthma further highlighted this trend, with 4%–17% of patients reported to experience anxiety and depression.^38^^,^^39^ WRA impedes the QoL of patients across various domains, including physical, psychological, economic, social, and treatment burdens. In particular, previous studies have presented the negative impact of WRA on QoL, in which patients with WRA were reported to have worse self-rated health, physical health, mental health, and activity limitation.^40^ Our findings highlight the profound influence of WRA on the QoL and psychological health of patients with severe asthma, accentuating the importance of integrating the detection of work-related exacerbations into the evaluation of severe asthma. By merging this with the assessment of psychological distress, a more encompassing therapeutic strategy that optimally enhances clinical outcomes could be ensured.

In the present study, patients with severe asthma and WRA did not have clinically more severe disease than those without WRA in terms of AE (steroid burst and unscheduled healthcare use due to asthma); this is in contrast to the findings of previous studies that have shown that patients with WRA or OA had higher asthma severity than those without WRA.^41^ Although WRA is a prominent occupational disease, its clinical characteristics, particularly in the context of severe asthma, remain under-researched compared to those of general asthma. In asthmatic populations, a recent study reported that patients with WRA had worse socioeconomic status, asthma control, QoL, and psychological status than those without.^42^ Another study demonstrated that patients with WRA had more AEs than those without.^41^ Our study focused on patients with severe asthma and found that most clinical and inflammatory asthma profiles were similar between the WRA and non-WRA groups at baseline. We speculate that the pathophysiology of WRA, in which various allergens, sensitizers, and irritants are involved, could have contributed to the similarities in inflammatory profiles. In addition, the non-WRA group consisted of patients with other phenotypes of severe asthma, including those with higher disease severity than the WRA group. A similar history of asthma between the groups is considered another contributing factor to comparable disease severity. A prospective multicenter study described the severity of OA as being related to the duration of symptoms before diagnosis but not to age, duration of exposure before symptoms, molecular weight of causative agents, or atopy.^43^ However, in this study, the severity of OA was defined by FEV1 and provocation dose of methacholine causing a 20% fall in FEV1, but AE or SABA use was not reflected in determining severe OA. A more recent study found that severe OA was associated with a longer duration of OA, persistent exposure to the causal agent, a low educational level, childhood asthma, and sputum production.^14^ The paucity of research on WRA in patients with severe asthma underscores the need for rigorous studies to elucidate the unique clinical features of WRA in this population, which could subsequently inform both clinical practice and occupational health policies.

This study has several limitations that should be acknowledged. First, the determination of WRA was primarily based on patients' responses to a questionnaire rather than a direct physician's diagnosis. In addition, we did not employ a bronchoprovocation test to validate the presence of WRA. Moreover, the patients were not categorized on the basis of their ongoing exposure to workplace materials that might induce or exacerbate their asthma. However, it is worth noting that while OA has specific diagnostic tests, the diagnosis of WRA is typically based on clinical judgment derived from patients' statements; therefore, the diagnosis of WRA in this study can still be deemed to be reasonably reliable. The second limitation pertains to the granularity of our analysis, in that we did not undertake a detailed examination by further categorizing WRA into OA and WEA. Such classification may have provided deeper insights into the nature and triggers of the disease. While OA include both sensitizer-induced asthma and irritant-induced asthma, we did not analyze these subtypes in this study. Another limitation is the cross-sectional design of this study. Data were collected only at the point of initial patient enrollment; therefore, longitudinal follow-up data were absent and continued exposure to the causal agent was not assessed. Including patients who may have ceased exposure to the triggering agent in the WRA group may have led to an underestimation of asthma severity. Longitudinal analysis is imperative to provide a more comprehensive understanding of asthma severity and associated clinical outcomes. Despite these limitations, this research holds significance as it represents the first study to investigate the prevalence of WRA among patients with severe asthma. These findings also shed light on the clinical attributes of severe asthmatics with WRA compared to their counterparts without WRA. The results underline the need for meticulously designed future studies to further elucidate the clinical and pathophysiologic implications of WRA in the context of severe asthma.

Our findings indicate that all age groups with severe asthma have a high incidence of WRA. Individuals with severe asthma who experience WRA are more likely to experience anxiety and depression and have a lower QoL than those without WRA.

## Abbreviations

AE, Asthma exacerbation; WRA, Work-related asthma; QoL, Quality of life; OA, Occupational asthma; WEA, Work-exacerbated asthma; KoSAR, Korean Severe Asthma Registry; ICS, Inhaled corticosteroid; LABA, Long-acting β_2_-agonist; GINA, Global Initiative for Asthma; CS, Corticosteroid; OCS, Oral corticosteroid; BMI, Body mass index; FEV1, Forced expiratory volume in 1 s; FVC, Forced vital capacity; FeNO, Fraction of exhaled nitric oxide; ACT, Asthma Control Test; QLQAKA, Quality of Life Questionnaire in Adult Korean Asthmatics; LTRA, Leukotriene receptor antagonist; SABA, Short-acting β_2_-agonist

## Funding

This research was supported by the 10.13039/501100003653Korea National Institute of Health research project (project No. 2022-ER1205-00).

## Availability of data and materials

The authors confirm that the data supporting the findings of this study are available within the article and its supplementary materials.

## Ethics

This study was approved by the Institutional Review Board of each participating hospital, and informed consent was obtained from the study participants (IRB number: AJOUIRB-OBS-2022-171).

## Author's consent for publication

All the authors reviewed the final draft and provided consent for publication.

## Declaration of competing of interest

There are no financial or other issues that might lead to conflicts of interest.
